# The Effectiveness of Emotionally Focused Couples Therapy on Sexual Satisfaction and Marital Adjustment of Infertile Couples with Marital Conflicts 

**DOI:** 10.22074/ijfs.2015.4556

**Published:** 2015-10-31

**Authors:** Ali Akbar Soleimani, Maryam Najafi, Khodabakhsh Ahmadi, Nasirudin Javidi, Elnaz Hoseini Kamkar, Mohamad Mahboubi

**Affiliations:** 1 Department of Psychology, University of Science and Culture, Tehran, Iran; 2 Behavioral Sciences Research Center, Baqiyatallah University of Medical Sciences, Tehran, Iran; 3 Abadan College of Medical Sciences and Health Services, Abadan, Iran

**Keywords:** Couples, Therapy, Adjustment, Sexual, Satisfaction

## Abstract

**Background:**

The purpose of this investigation is to determine the efficacy of emotionally focused couples therapy (EFT-C) on enhancement of marital adjustment in infertile couples.

**Materials and Methods:**

This was a semi-experimental study with a pre- and post-test design. We selected 30 infertile couples (60 subjects) by purposive sampling. Couples were randomly assigned to two groups, sample and control. Each group consisted of 15 couples who had marital maladjustment and low sexual satisfaction. Couples answered the marital adjustment and sexual satisfaction questionnaires at baseline after which the sample group received 10 sessions of EFT-C.

**Results:**

Results of pre-test and post-test showed that EFT-C significantly impacted marital adjustment and sexual satisfaction.

**Conclusion:**

EFT-C had a significant effect on enhancement of satisfaction, cohesion and affectional expression. This approach impacted physical and emotional sexual satisfaction of infertile couples.

## Introduction

As marital life begins, couples expect to have children because with the birth of a baby, life will head toward another path. In contrast, in the case of infertility, serious psychological problems may be faced by partners (whether male or female) (1). Although in recent years infertile couples have greater opportunities to bear offspring due to advances in medical science and various fertility methods (2), this situation, as a tense crisis, influences different life aspects of infertile couples (3). For most people, children are the meaning of life and constitute an important part of their identity. Multiple researches on infertile couples that yearn for biological babies have shown that these couples experience tension in a deep, distressed manner (4). The medical definition of infertility is as follows: after one year of usual sexual activity without contraception if the woman is either not pregnant or fails to become pregnant, she is considered infertile (5).

Infertility brings about detrimental psychological effects (6) that include reduced self-confidence, disordered self-image, and impairment of masculine and feminine identities (7). Some researchers believe that infertility is a challenging experience which leads to problems in marital life (8).

Marital adjustment is a changing process that includes four aspects of couples’ performance as a joint life: i. Marital life satisfaction, ii. Commitment to marriage, iii. Agreement and unanimity in marital life, and iv. Manifestation and expression of couples’ emotions and feelings within the family (9). Couples who agree with each other are relatively satisfied with their marital life and express satisfaction with their partner’s personality habits, enjoy companionship with their family and friends, solve problems together, and are highly satisfied with their marital and sexual life (10). One of the biggest problems that influence one’s personal and social life is sexual satisfaction which plays a crucial role in personality development. Just like other fundamental motivations of human beings, sexual motivation and desire constitute an inevitable part of their biological, psychological, and social natures. The quality with which this a biological need is met plays a very important role in personal and social health, thereby achieving relaxation and comfort (11). Couples’ degree of satisfaction with sexual relations and ability to take pleasure in and give pleasure to one another is called sexual satisfaction. Gratification from sexual relations is one of the important factors of marital life satisfaction, and those with greater sexual satisfaction considerably report a better life related to those without sexual satisfaction (12).

A desirable sexual relation can increase the possibility of fertility. Furthermore, infertility, in turn, can influence couples’ sexual relations. It is thought that psychosexual disorders in infertile couples are more prevalent than other couples (13). Infertility leads to increased sexual disorders, maladjustment, reduced sexual satisfaction and sexual activity times (14). Infertility influences one’s sense of self, sexual identity, self-confidence, and body-image, it inevitably impacts sexual relations, the importance of sexual relations, and sexual desire along with satisfaction (15). Researches show that men and women react differently toward infertility.

Gender is an important factor in assessing differences between men and women regarding the stress of infertility and sexual satisfaction. Duration of infertility and the reason and type of infertility are among important factors of sexual and marital satisfaction of infertile couples. Besides age, marriage length, education, income, and social class have been stated in different resources as factors that influence infertile couples’ sexual and marital satisfaction (16). Infertility leads to couples’ reduced sexual satisfactions (17). Anxiety and loss of self-confidence, shame, and depression that result from infertility harm infertile people’s sexual performance. Moreover, diagnosis, examination and treatment of infertility affect their sexual satisfaction (18). Dyer et al. (19) have reported that dissatisfaction with sexual relations, stress and planning for sexual relations, and lack of sexual selfesteem in infertile women have the highest impact on their sexual satisfaction. Additionally, since women have reported that they only think about having a baby while having a sexual relation, thus this concern leads to increased stress (15).

Infertile couples experience tremendous stress at the first phase of treatment regarding their sexual activity, especially sexual desire and sexual arousal. Women suffer more than men. Therefore, doctors must pay attention to sexual problems of couples in order to prevent a vicious cycle which may decrease the possibility of pregnancy and cause permanent disorder to their sexual relations (20).

The relationship between infertility and sexuality can be seen from two angles: i. Infertility as a cause or ii. Infertility as a result of sexual dysfunction. However, sexual disorders are considered as minor reasons for infertility as approximately 5% of all infertile cases result from sexual disorders (21). In addition, men’s sexual disorders include chronic erection dysfunctions and non-ejaculation while the only sexual disorder in women in terms of infertility is vaginismus (22). Since the label of infertility is worrisome, sexual intercourse can lose its spontaneity (23).

Examination and treatment of infertility in traditional social interactions leads to a high level of psychological distress that directly affects couples’ marital and sexual relations (14, 24-26). The clinical effect and impact of this disease on couples’ overall health, daily performance, social interaction and marital relations, and quality of life is partly underestimated (24).

In the examination of an infertile couple, it is highly important to note that emotional factors, as sexual disorders attributed to emotional factors, may lead to infertility. Emotions have a key role in the infertile couples’ relations and must be given special attention. Therefore, one can use emotionally focused couples therapy (EFT-C) that consists of 9-20 short-term, structured sessions. This therapy is both a branch of couple’s therapy and regards emotions as a treatment axis. This treatment addresses relational disorders and maladjustment and encourages people to talk about and discuss their emotions. From the EFT-C point of view, the center of marital distress is created and continued by negative affection and attachment injuries (27).

In EFT-C, it is assumed that conflict in marital life occurs when spouses fail to meet each other’s attachment needs for security, safety, and satisfaction. In other words, disturbed marital relations are suggestive of the couples’ failure in establishing relations associated with a safe attachment pattern. By not meeting each other’s attachment needs, these spouses experience secondary emotional responses such as anger, hostility, vengeance, or feelings of guilt.

Secondary emotional responses are also manifested in avoidant aggressive behaviors, which may ultimately lead to the creditor-creditor or avoidanceavoidance models. These inflexible interactional models which also fuel conflict occur repeatedly because spouses desperately want their genetic attachment needs to be met. Unfortunately, partners’ efforts to get the spouses’ attention are not made properly. Consequently, the partners are forced into relations that lead to continued failure of attachment needs (28). Accordingly, EFT-C seeks to solve couples’ problems by focusing on their emotional relationships. The change process of EFT-C has been specified in three phases that include: i. Prevention from development of a vicious cycle; ii. Reconstruction of interactional situations and iii. Consolidation and integration (29). Therefore, the present study attempts to explain whether EFT-C can increase the adjustment of conflicting infertile couples that suffer from low sexual satisfaction.

## Materials and Methods

This was a quasi-experimental study that used preand post-tests on two groups, control and sample.

To test the hypothesis of research, that is the rejection of null hypothesis (Ho) and the acceptance of (HA), values of 0.05 and 0.01 were used for alpha. In other words, for the level of significance, probability of error was determined to be less than P<0.05 or P<0.01 and if greater than 0.05 it was considered to be non-significant.

We assessed subjects’ demographics after which they were measured before conducting the independent variable (EFT-C) as a pretest. Then, we administered EFT-C to each couple in the experimental group over a period of 10, 120 minutes sessions in accordance with Johnson’s model (27). Subjects were subsequently re-evaluated (post-test).

The statistical population of the study included couples who visited infertility centers during 2013 and were identified by obstetricians and gynecologists as infertile. Couples were living with each other for at least 10 years. Individuals (men and women) completed the marital adjustment and sexual satisfaction questionnaires.

In this research, purposive sampling was used whereby 30 couples (60 subjects) were selected and randomly assigned to two groups, sample and control. Each group consisted of 15 couples who had marital maladjustment and low sexual satisfaction. Inclusion criteria for the study were marital conflict and low sexual satisfaction in infertile couples, interest in couples therapy sessions, present for all treatment sessions, having at least a high school level of education, no significant acute mental- physical disorders as self-reported in the demographic characteristics questionnaire, and loss of fertility after 10 years of marital life. Exclusion criteria included lack of marital conflicts and high sexual satisfaction in infertile couples, non-participation in all stages of measurement and intervention, having a significant acute mental-physical disorder through the self-reported demographic characteristics questionnaire, primary school level of education, and infertility less than 10 years. All participants expressed consent to participate.

In order to analyze data, we used descriptive statistical methods that measured the mean, maximum and minimum standard deviation. Analysis of covariance was adopted using SPSS version 18 in the inferential section.

### Research tools

#### Questionnaire of demographic characteristics

This inventory included factors of age, sex, educational level, occupation, income level, and cause of infertility, period of infertility, quantity of surgeries, date of last surgery, history of attending psychological or counseling sessions, as well as histories of any chronic physical or psychological disorders (30).

#### Spanier’s Dyadic Adjustment scale

This scale is constituted of 32 questions based on a Likert scale of responding which measures the total score of marital adjustment in a range of 0 to 15. Individuals who score 101 or less according to Spanier, are deemed as maladjusted and those with higher scores are supposed to be well-adjusted. In a study by Hasan shahi (28), well-adjusted couples had an average score of 114.7 ± 17.8 while the average score for maladjusted couples was 70.7 ± 23.8. Spanier categorized the data into four subscales of marital satisfaction, dyadic consensus, dyadic cohesion, and affectional expression with evaluated validities of 0.94, 0.90, 0.81 and 0.73 respectively. The entire scale had a validity of 0.96. Reliability was estimated to be 0.86 according to Pearson’s correlation coefficients between Locke-Wallace Marital Adjustment Scale and Spanier’s scale (30).

#### Index of Sexual Satisfaction

This scale was developed by Hudson et al. and revised by Javidi (15). In 1981 it contains 25 questions with a 5-point Likert response scale (1=never, 2=rarely, 3=sometimes, 4=most often, 5=always). This scale evaluates sexual satisfaction in two aspects of physical satisfaction and emotional satisfaction. The dimension of physical satisfaction includes sexual behaviors and sexuality whereas the emotional aspect includes intimacy and quality of sexual relations. Hudson believes that this scale assesses sexual satisfaction through the intensity and extent of sexual components. Internal consistency of this test by Cronbach’s alpha coefficient was calculated to be 0.92. Studies have indicated that this questionnaire is significantly related to scales designed to measure similar constructs. The correlation coefficient of this scale with the Marital Satisfaction questionnaire was 66% (31). This was the first time this scale was used in Iran. Hence, we assessed the psychometric properties from two aspects, reliability and validity. The alpha value for the entire scale was calculated to be 0.88 and for emotional satisfaction this value was 0.90. The alpha value of the sexual behavior subscale was 0.84, for sexuality it was 0.78, sexual intimacy was 0.87, and quality of sexual relations was 0.74. The reliability coefficient for sexual satisfaction by the method of split-half was calculated to be 0.85 and the Spearman revised coefficient was 0.92 (32). Table 1 points out the treatment protocol used in this study, this protocol is emotionally-focused therapeutic approach, which have been provided to the couple during 10 sessions.

### Ethical consideration

In order to observe ethical considerations, in this study, the researchers made a great importance to the confidentiality and preserving the couples’ dignity. In addition, since the emotionally-focused treatment training has been effective in experiment group, the researchers also carried out such training for members of the control group in their training sessions after the completion of their work.

## Results

The demographic description of the sample is provided in table 2. There were 30 participants of both genders. The maximum and minimum frequencies in terms of educational level were 19 (31.7%) individuals with diplomas and 10 (11.7%) people with secondary school certificates. The average age of participants was 33.8 ± 5.03 years.

First, we used the Kolmogorov-Smirnov test. The data was approved as normal for all variables (P>0.05). There was no significant difference between groups in the pre-test subscales of marital adjustment and sexual satisfaction.

Table 2 shows no significant difference between groups in marital adjustment and sexual satisfaction (P>0.05). Therefore both groups were the same at the pre-test stage. According to table 2, it could be inferred that no significant difference existed between groups in the pre-test sexual satisfaction subscales (P>0.05).

According to the covariance analysis test results in the dimensions of marital adjustment, there was a significant difference between the pre- and posttests in terms of couples satisfaction, couples correlation, couples agreement, expression of love, and sexual satisfaction (physical and emotional, P<0.001).

In order to compare average sexual satisfaction of the sample group with the control group at the pretest stage, we statistically compared the two independent means. The Kolmogorov-Smirnov test was also employed to assess normal distribution of the variable of sexual satisfaction which, according to the results, was confirmed (P=0.599 and Z K-S=-0.526). Thus, comparison of two population means is practicable. Table 3 shows the results of ANCOVA of marital adjustment and sexual satisfaction subscales in couples.

**Table 1:** Johnson’s protocol of emotionally focused therapy (EFT-C) for couples suffering from infertility(30)


Step	Session	To do

1 Identification	1	Collect general information about the couple; introduce the therapist to the partners; investigate grounds and expectations of participation; define the method of EFT-C in addition to concepts of infertility, conflict, marital adjustment, sexual satisfaction, and life quality; ask the couple for their opinion on the method and concepts; identify negative cycles; assess couple’s way of dealing with issues; discover attachment blocks as well as personal and interpersonal tensions; evaluate status of marital relationship, sexual satisfaction and quality of life.
		Task: Pay heed to positive and negative emotions such as joy, happiness, anger, hate, sadness, jealousy, anxiety, etc.
	2	Appoint a separate session for each partner to discover significant events, and information that is not feasible to discuss in the presence of the other, such as commitment to marriage, extramarital relationship, exporter attachment trauma; assess the fear of revelation.
		Task: Pay attention to your partner’s cycle of interaction.
2 Change	3	Ascertain interaction patterns and ease acceptance of the experienced emotion; discern every partner’s fears of insecure attachment; help each partner with openness and selfdisclosure, continue the therapy.
		Task: Discern pure emotions, thoughts, and sentiment.
	4	Restructure the bond through clarification of key emotional reactions; widen emotional experience of each spouse to create new ways of interaction, partners should accept new patterns of behavior.
		Task: Express pure emotions and sentiments.
	5	Task: Deepen the relationship by recognizing recently developed needs of attachment, improve personal health and relationship status, express pure emotions and sentiments
3 Stabilization	6	Establish a safe therapeutic alliance, develop new ways of interaction; promote acceptance of the other, discover deep-seated fears and express needs and wants.
	7	Restructure the emotional experiences of the couple, clear the needs and wants of each partner.
		Task: Underline strengths and weaknesses.
	8	Support couple in finding new solutions to past problems; change problematic manners of behavior, facilitate steps the couple can take to invest in their responsive and accessible positions, sync the inner feelings and concepts to the relationship, encourage positive reaction.
		Task: Find new solutions to past problems.
	9	Take advantage of therapeutic achievements within daily life to consolidate intimacy, keep going with the therapy and its direction, create secure attachment, discern and support constructive patterns of interaction; help the couple shape a story about their future together.
		Task: Practice the techniques in daily life.
	10	Ease the end of the treatment, keep the way of therapeutic changes, draw a comparison between the past and present cycles of interaction, keep on emotional involvement to the deepest status of the relationship.


**Table 2:** Pre-test comparison of the groups in the subscales of marital adjustment and quality of life


Subscales	Group	Mean	Standard deviation	t^*^	df^**^	P value^***^

Satisfaction of dyadic	Control	22.18	4.34	0.813	58	0.425
	Sample	21.29	4.25			
Cohesion of dyadic	Control	7.94	2.05	1.087	58	0.283
	Sample	7.35	2.17			
Consensus of dyadic	Control	24.21	5.91	1.493	58	0.146
	Sample	21.81	6.53			
Affectional expression	Control	4.67	1.30	0.785	58	0.432
	Sample	4.40	1.33			
Physical sexual satisfaction	Control	30.06	7.38	0.99	58	58
	Sample	28.20	7.20			
Emotional sexual satisfaction	Control	21.20	8.30	2.269	58	0.027
	Sample	16.83	6.49			


^*^; Paired t test, ^**^; Degrees of freedom and ^***^; Probability of rejecting the null hypothesis.

**Table 3:** ANCOVA of marital adjustment and sexual satisfaction subscales in couples


Aspects	Freedom	Mean square		F-value		P value		Effect size		Statistical power	
	Pretest	Pretest	Group membership	Pretest	Group membership	Pretest	Group membership	Pretest	Group membership	Pretest	Group membership

Satisfaction of Dyadic	1	141.82	5138.84	10.12	363.96	0.002	0.001	0.151	0.87	0.88	1
cohesion of Dyadic	1	54.07	62.478	15.38	15.86	0.001	0.001	0.212	0.93	0.96	1
consensus of Dyadic	1	427.14	16503.14	13.21	542.54	0.001	0.001	0.19	0.90	0.92	1
Affectional expression	1	10.126	703.516	8.14	565.79	0.006	0.001	0.125	0.91	0.80	1
Physical sexual satisfaction	1	82.338	18769.06	1.16	263.47	0.287	0.001	0.002	0.82	0.18	1
Emotional sexual satisfaction	1	141.48	22023.44	50.3	545.83	0.066	0.001	0.058	0.90	0.45	1


According to the results from Levene’s test, equality of the variances in the sample and control groups was corroborated (P>0.05). Therefore, use of covariance analysis was permitted. The results of covariance analysis related to comparison of mean scores of studied dimensions in the sample and control groups are shown below.

As table 2 shows, there was a statistically significant difference between the groups (P<0.001). Thus, EFT-C positively affected sexual satisfaction and marital adjustment in infertile couples. The effective percentage of intervention was: dyadic satisfaction (86%), dyadic cohesion (92%), dyadic consensus (90%), affectional expression (90%), physical sexual satisfaction (82%) and emotional sexual satisfaction (90%0, which indicated the efficacy of EFT-C.

## Discussion

Findings of the present study were consistent with related studies. In different studies (32-34) it was found that infertile couples did not have a clear sign and regardless of men’s sexual role, obtained a higher score in the sexual satisfaction, sexuality, and orgasm items of the questionnaire. A comparison of the results of Experimental and control groups in terms of sexual arousal, satisfaction, lust, and orgasm, suggested that the experimental group was weaker than the fertile control group. Sexual life of infertile couples was poor due to infertility. In his study entitled "Sexual Disorders in Infertile Couples", Wischmann (35) concluded that sexual dysfunction, as the factor of reluctance to have children, was relatively abnormal. Instead, temporary sexual disorders in couples with infertility affected women more than men due to diagnostic complications and pharmacotherapy. Counseling for couples with unfulfilled desires to have children should include clear, appropriate clarification of sexual and gender disorders.

Findings of this research corresponded with the results of a study by Greil et al. (36) as well as another research by Martins et al. (37). Greil et al. (36) declared that EFT could have significant positive and constructive effects on the relationship of couples and their satisfaction with life which might have declined due to infertility. Greil et al., through a meta-analysis, stated that a variety of psychological interventions could improve both life quality and relationship of infertile couples. The single difference between these methods was the size of the effect. Couples therapy surpassed the other interventions in this respect. This achievement could be attributed to the fact that couples therapy during the intervention points out at the same time.

Tie and Poulsen believed that EFT was influential in the promotion of couples’ marital adjustment. After intervention, the relationship of couples improved and they expressed increased satisfaction from their spouses when compared to the past. Marital disputes significantly decreased and an upturn was observed in marital adjustment. The results of Tie and Poulsen’s research have been confirmed by the present study. Dyadic consensus is one the aspects of marital adjustment. Increase in this area denotes growth of dyadic consensus (38).

Bodur et al. (39) measured the effect of marital adjustment in infertile couples on stress related to infertility. This study included 104 couples with primary and/or secondary infertility and 44 fertile couples as the control group. Women in infertile groups reported more psychological symptoms and decreased marital adjustment than men in infertile groups, but there was no significant difference between partners in the control group regarding the aforementioned parameters. In general, infertile couples had decreased marital adjustment and increased depression and anxiety levels. Nevertheless, if infertile couples were mentally supported and received service from social systems, their marital adjustment would increase and psychological symptoms disappear.

Barani Ganth et al. (40) reported that fertile couples were more satisfied with their marital life than infertile. The effect of infertility on women’s satisfaction appeared more concerning compared to men. Infertility in women has been shown to lead to a life with low marital satisfaction.

Jalil and Muazzam (41) found a significant relationship between emotional intelligence and marital satisfaction in both groups. A comparison of both groups showed that fertile women with higher emotional intelligent had greater levels of marital adjustment. On the contrary, infertile women with lower emotional intelligent suffered from poorer marital satisfaction.

Moura-Ramos et al. (42) showed that contextual factors such as socioeconomic status and urban or rural residence impacted affective anxiety in infertile couples. An Investigation into marital relationships in infertile couples showed that male infertility did not any negative effect on the marital relationship.

In Austria, Drosdzol and Skrzpulec (43) evaluated sexual and marital Interactive responses in infertile couples which showed that marital relations of infertile women were less than fertile women. As a result, the former were more prone to marital disorders than fertile women.

Mira (44) investigated the effect of EFT-C on knowledge, competence, emotional processing, and self-compassion of 76 participants. The results showed that knowledge and competence of participants in the experimental group increased as a result of therapy. He also stated that a significant relationship existed between competence and the emotional process of individuals as well as self-compassion. Javidi et al. (45) investigated the long-term effect of EFT-C on knowledge, competence, self-compassion, and secure attachment. Results showed the effectiveness of EFT-C in increasing knowledge, competence, self-compassion, secure attachment, and personal interactions.

Pinto-Gouveia et al. (46) investigated the efficacy of protective emotion-regulation training toward adjustment in infertile patients. They reported that infertile couples had problems with expression of emotions and mutual understanding of their partners’ feelings which were notably resolved by training in emotion-regulation strategies. After intervention, couples had great success in affectional give and take with improvement in their marital adjustment (46). Javidi et al. (47) indicated that EFT-C could significantly increase sexual satisfaction of couples.

Finally, it can be said that EFT-C trains couples to correct their behavior through increasing security and support, availability, response to the spouse’s need and creating safe behaviors, methods of increasing intimacy and relationships, learning proper communication skills, and establishment of desirable sexual relations.

This study has regarded sexual satisfaction as an important factor for controlling daily emotions. In explaining the reason for the increase in couples’ sexual satisfaction due to EFT-C, it can be said that this therapy teaches couples to reveal the important problems of their life to their spouses, receive a positive response from spouses. In addition, they can also increase their verbal and non-verbal interactions, show sexual self-expression including touching, hugging, and kissing, express their thoughts, feelings, needs and tendencies, and have more physical closeness. Considering that sexual relation is among the most important matters in marital life and acts as the emotional barometer in relations, it can reflect couples’ satisfaction with other aspects of the relationship. Thus, it is a good scale of the overall health of couples’ relations.

Use of this treatment plan can help to increase couples’ sexual satisfaction and intimacy, leading to improved overall relations for couples.

## Conclusion

This treatment approach can increase adjustment and sexual satisfaction in couples. Results from this research can create a clear and practical outlook for counselors, psychotherapists, and family therapists. The results can provide couples with desirable applied and empirical guidance for creating self-esteem and detection, and revision of inconsistency in their way of giving messages to each other, their communication patterns and, as a whole, human growth which consequently reduces marital conflicts and increases adjustment. The results of the emotional sexual satisfaction dimension were consistent with those reported by Huppelschoten et al. (48).

EFT-C, through concentrating on the emotional relationship, manages to solve couples’ problems. Accordingly, marital disputes that result from emotional problems and insecure attachment can be addressed by EFT-C. Sexual dissatisfaction generally appears in the form of complaining, blame, or rebuke and extremely endangers attachment. The sexual relationship is more than intercourse and is a way to achieve harmony and create positive emotions which thereby strengthens attachment (49). Application of this therapeutic plan can promote sexual satisfaction of couples and their intimacy.

By taking into consideration the effects of EFT-C on marital adjustment and sexual satisfaction, we propose that additional research should investigate which technique from the EFT-C approach has the highest level of effectiveness.

As infertility has negative effects on marital satisfaction, it is recommended that necessary training be provided in fertility clinics to lessen social and psychological stress of clients and increase their marital adjustment. Since familial disputes are the most important cause for separation between infertile couples, training in marital relationships can prevent couples from divorcing and instead, reinforce their family’s foundation.

Constraints of the study included the number of questionnaires and large numbers of questions which made the respondents tired. Additionally there was no follow-up due to participant refusal.
